# The Ectopic Expression of CaRop1 Modulates the Response of Tobacco Plants to *Ralstonia solanacearum* and Aphids

**DOI:** 10.3389/fpls.2016.01177

**Published:** 2016-08-08

**Authors:** Ailian Qiu, Zhiqin Liu, Jiazhi Li, Yanshen Chen, Deyi Guan, Shuilin He

**Affiliations:** ^1^College of Life Science, Fujian Agriculture and Forestry University, FuzhouChina; ^2^Key Laboratory of Crop Genetics and Breeding and Comprehensive Utilization, Ministry of Education/Fujian Agriculture and Forestry University, FuzhouChina; ^3^College of Crop Science, Fujian Agriculture and Forestry University, FuzhouChina

**Keywords:** *CaRop1*, *Capsicum annuum*, *Ralstonia solanacearum*, aphid, JA/ET-mediated signaling

## Abstract

In plants, Rho-related GTPases (Rops) are versatile molecular switches that regulate various biological processes, although their exact roles are not fully understood. Herein, we provide evidence that the ectopic expression of a Rop derived from *Capsicum annuum*, designated CaRop1, in tobacco plants modulates the response of these plants to *Ralstonia solanacearum* or aphid attack. The deduced amino acid sequence of CaRop1 harbors a conserved Rho domain and is highly homologous to Rops of other plant species. Transient expression of a CaRop1-GFP fusion protein in *Nicotiana benthamiana* leaf epidermal cells revealed localization of the GFP signal to the plasma membrane, cytoplasm, and nucleus. Overexpression (OE) of the wild-type CaRop1 or its dominant-negative mutant (DN-CaRop1) conferred substantial resistance to *R. solanacearum* infection and aphid attack, and this effect was accompanied by enhanced transcriptional expression of the hypersensitive-reaction marker gene *HSR201*; the jasmonic acid (JA)-responsive *PR1b* and *LOX1*; the insect resistance-associated *NtPI-I*, *NtPI-II*, and *NtTPI*; the ethylene (ET) production-associated *NtACS1*; and *NPK1*, a mitogen-activated protein kinase kinase kinase (MAPKKK) that interferes with N-, Bs2-, and Rx-mediated disease resistance. In contrast, OE of the constitutively active mutant of CaRop1(CA-CaRop1) enhanced susceptibility of the transgenic tobacco plants to *R. solanacearum* infection and aphid attack and downregulated or sustained the expression of *HSR201*, *PR1b*, *NPK1*, *NtACS1*, *NtPI-I*, *NtPI-II*, and *NtTPI*. These results collectively suggest that CaRop1 acts as a signaling switch in the crosstalk between Solanaceaes’s response to *R. solanacearum* infection and aphid attack possibly via JA/ET-mediated signaling machinery.

## Introduction

In their natural habitats, plants are frequently exposed to attack by a wide variety of biological stresses, such as microbial pathogens and herbivorous predators. The evolutionary arms race between plants and their enemies has provided plants with a highly sophisticated defense system to perceive and discriminate invaders and to tailor their defense responses against specific invaders, thereby attaining higher fitness and survival rates. This defense system is regulated by complex signaling networks. Examples include calcium signaling; G-protein signaling; stimulation by phytohormones, specifically salicylic acid (SA), jasmonic acid (JA), and ethylene (ET); phosphorylation mediated by various kinases; and a burst of reactive oxygen species (ROS). Various transcription factors, such as WRKY and MYC2, have been frequently found to be shared by response pathways directed against plant pathogens/insects (Bodenhausen and Reymond, 2007; Dombrecht et al., 2007; Maffei et al., 2007; Pozo et al., 2008; Nascimento and Fett-Neto, 2010; Atamian et al., 2012; Kutyniok and Muller, 2012; Cheng et al., 2013; Dang et al., 2013, 2014; Kazan and Manners, 2013; Wang et al., 2013). The signaling pathways mediated by distinct components may interact with each other differently depending on the conditions. For example, SA- and JA-dependent signaling pathways may interact either synergistically or antagonistically, depending on the relative concentration of activating hormones (Mur et al., 2006). Components may act as key nodes in cross-communication signaling networks between plant responses and pathogen infection and insect attack (Bostock, 2005). In addition, the kinetics of the signaling may vary greatly in both quantity and timing, either in different plants or in the same plants attacked by different pathogens or insects in order to optimize the response against a single attacker (De Vos et al., 2005; Xie et al., 2012). Although plant responses to herbivores and pathogens has been the focus of much work over the past decades, the signaling pathways of plant responses to these challenges and their molecular and pathway crosstalk is still not well understood, especially with respect to early signaling cascades.

The Rho-related GTPases of plants (Rops) family, also known as the Rac family, belongs to the Ras superfamily of small GTPases (Zheng and Yang, 2000). Like other small GTPases, Rops act as versatile molecular switches by cycling between GDP-bound inactive and GTP-bound active forms in cells and are regulated by a set of guanine nucleotide exchange factors, GTPase-activating proteins (GAPs), and guanine nucleotide dissociation inhibitors (GDIs; Eklund et al., 2010). Loss-of-function mutation of these proteins are likely to induce no clear phenotypic effects due to additional redundant proteins. For this reason, Rops GTPase mutants that are constitutively active (CA) or dominant-negative (DN) due to the presence of conserved point mutations have become important tools for studying Rop proteins. In fact, Rop proteins in CA- and DN-transgenic lines of *Arabidopsis* and rice have been studied with these tools (Zheng and Yang, 2000; Ono et al., 2001; Fu et al., 2002; Tao et al., 2002; Zheng et al., 2002; Poraty-Gavra et al., 2013). Rops are important in a wide variety of physiological plant processes, such as polar morphogenesis of tip-growing cells in pollen tubes and root hairs (Kost, 2008; Lee and Yang, 2008), polar morphogenesis of leaf epidermal cells (Yang, 2008; Singh et al., 2013), branching of trichomes and root hairs (Duan et al., 2010; Singh et al., 2013), lignin and secondary cell wall synthesis (Kawasaki et al., 2006; Foucart et al., 2009), asymmetric cell division (Humphries et al., 2011), regulation of cytoskeletal dynamics (Mucha et al., 2011), cell expansion and stomata development (Pathuri et al., 2009), and abscisic acid (ABA)-mediated stomatal closure (Li and Liu, 2012). These proteins have also been implicated in adaptation of plants to various environmental cues, including pathogen infection (Agrawal et al., 2003; Thao et al., 2007; Chen et al., 2010a; Huesmann et al., 2011, 2012; Kim et al., 2012; Poraty-Gavra et al., 2013), hypoxia (Steffens and Sauter, 2010), salt-stress signaling (Luo et al., 2006), and drought stress (Li et al., 2012). Moreover, several biological processes have been found to involve specific Rops. For example, Atrop6 (DN) plants are small and have multiple inflorescence stems, twisted leaves, deformed leaf epidermis pavement cells, and differentially organized cytoskeleton. A SA-mediated defense response in these plants was conferred by overexpression (OE) of DN-rop6 (Poraty-Gavra et al., 2013). In addition, ABA-mediated responses affected by Rop11 include seed germination, seedling growth, stomatal closure, induction of ABA-responsive genes, and plant response to drought stress (Li et al., 2012); however, the functions of Rop family members in different plant species have not been full characterized.

Pepper (*Capsicum annuum*) is an important agricultural crop worldwide. As with typical *Solanaceae* sp., pepper plants are susceptible to many soil-borne pathogens and are, therefore, sensitive to continuous cropping. Of particular note is the susceptibility of this species to *Phytophthora capsici* and *Ralstonia solanacearum*, the two most important causal agents of pepper diseases worldwide. Aphids are notorious pests in pepper and cause heavy losses, either by direct attack or indirect damage as a vector for cytopathic viruses, including tobacco mosaic virus (TMV). Studies of interaction between pepper and pathogen/pest may contribute to development of new pepper cultivars with improved resistance to these biotic stresses and offer effective means for long-lasting control of pepper diseases and/or pests. Until now, no Rop/Rac gene has been cloned and functionally characterized in pepper disease or pest resistance. In the present study, we demonstrate that ectopic expression of the DN or CA mutants of CaRop1, a Rop from *C. annuum*, modulated the response of tobacco plants to *R. solanacearum* and aphid attack possibly via JA/ET-mediated signaling machinery.

## Materials and Methods

### Plant Materials and Growth Conditions

Pepper plants (*C. annuum)* from the genetically stable inbred line p120 were provided by the pepper breeding group at the Fujian Agriculture and Forestry University (FAFU). The seeds of *Nicotiana benthamiana* and the *Nicotiana tabacum* cultivar K326 (including its transgenic lines) were provided by Professor Fang of the College of Plant Protection at FAFU. *C. annuum* or *N. benthamiana* seeds were sown in a soil mix of peat moss and Perlite (2:1, v/v) in plastic pots. Tobacco seeds were surface sterilized in 75% alcohol for 30 s, incubated in 10% H_2_O_2_ for 10 min, and then washed five times with sterile ddH_2_O. Transgenic tobacco seeds were placed on Murashige and Skoog medium (MS; Murashige and Skoog, 1962) supplemented with 75 mg L^-1^ kanamycin for 2–3 weeks, while wild-type tobacco seeds were placed on MS medium without supplement. Surviving tobacco seedlings were transferred into soil mix (peat moss:perlite [2:1, v/v]) in plastic pots. All seedlings were grown in a greenhouse under white fluorescent light (70 μmol photons m^-2^s^-1^, OSRAM, China) for 16 h d^-1^ at 25°C and 70% relative humidity.

### Isolation and Sequence Analysis of *CaRop1* cDNA

Full-length cDNAs of Rop GTPase genes from pepper were obtained by searching expressed sequence tags (ESTs) of *C. annuum* (taxid: 4072) with the sequence of AtRac1 as a query using TBLASTN^1^. Matching ESTs were assembled into contigs using DNAMAN software. Specific PCR primers were designed from a contig with high sequence similarity to *AtRac1*. Corresponding cDNA clones were screened from a cDNA library by a PCR-based 96-well screening method (Munroe et al., 1995). Positive clones (λTriplEx2) were converted to pTriplEx2 by excision *in vivo* according to the manufacturer’s protocol (Clontech, Mountain View, CA, USA). The pTriplEx2 samples were sent to TaKaRa (Dalian, China) for sequencing. The sequence data were analyzed using the BLAST program from NCBI^1^. Homology and conserved domains were compared against sequences of other plant species in GenBank using DNAMAN software as described previously (Zheng and Yang, 2000; Agrawal et al., 2003). A phylogenetic tree was constructed with MEGA5.0 software using the neighbor-joining method.

### Construction of *CaRop1* Mutants

**FIGURE 1:**
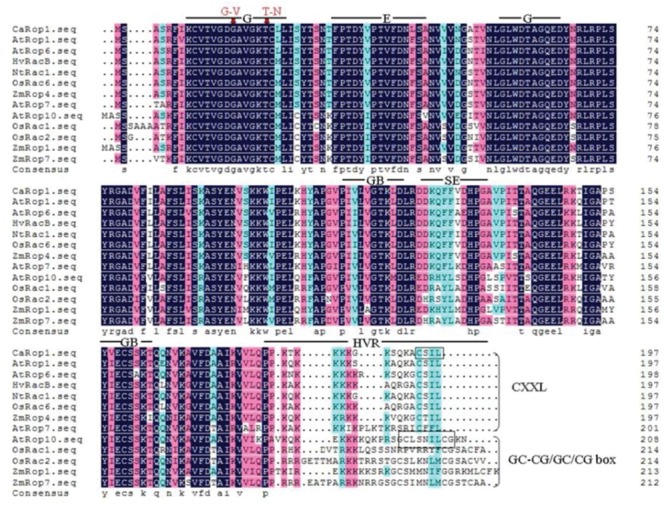
**Analysis of the deduced amino acid sequence of CaRop1 and comparison with homologous sequences from other monocot and dicotyledonous (dicot) species using DNAMAN5.0.** The high-, low-, and neutral-consensus amino acid residues are depicted in black, blue, and red colors, respectively. Bars indicate conserved functional domains of Rop/Rac family proteins. Abbreviations: G, GTPase domain; E, effector domain; GB, GDP/GTP-binding domain; SE, special effector loop (Rho insert region); HVR, C-terminal hypervariable region. To produce constitutively active (CA) and dominant-negative (DN) forms of *CaRop1*, the glycine (G) and threonine (T) residues marked with red arrows were changed to valine (V) and asparagine (N), respectively. The two types of Rops, based on the C-terminal motif, are indicated with a black frame and brace: the type-I Rops are characterized by the presence of a CXXL motif, and the type-II Rops lack this motif but contain a GC-CG/GC or CG box.

The CA and DN CaRop1 mutants were produced as described in previous studies by changing the glycine (G) and threonine (T) residues (marked by arrows in **Figure 1**) in the domain I to valine (V) and asparagine (N), respectively (Agrawal et al., 2003; Schallhart et al., 2012). An overlap-extension PCR protocol for site-directed mutagenesis was performed, and sequences around the point to be mutated (**Figure 1**) were designed as forward primers (CA-*CaRop1*-F, DN-*CaRop1*-F) and reverse primers (CA-*CaRop1*-R, DN-*CaRop1*-R). To produce CA*-CaRop1*, three rounds of PCR were performed. The first and second rounds of PCR were performed with the primer combinations *CaRop1*-F/CA-*CaRop1*-R and CA-*CaRop1*-F/*CaRop1*-R, respectively. The amplified products were mixed and used as the template for the third round of PCR, with the primer combination *CaRop1*-F/*CaRop1*-R. Similarly, to produce DN-*CaRop1*, the first and second rounds of PCR were performed with the primer combinations *CaRop1*-F/DN-*CaRop1*-R and DN-*CaRop1*-F/*CaRop1*-R, respectively, and the third round was carried out with the primer combination *CaRop1*-F/*CaRop1*-R (**Table 1**). The two amplified mutants were subcloned into pDONR207 entry vectors, according to the supplier’s instructions (Invitrogen, Carlsbad, CA, USA). The two mutants were confirmed by DNA sequencing.

**Table 1:** All primers used for PCR in this study.

Gene	Accession no.	Forward primer name and sequence (5′→3′, -F)	Reverse primer name and sequence (5′→3′, -R)
*CaRop1*	DQ257288	AAAAAGCAGGCTTTATGAGTGC TTCCAGGTT	AGAAAGCTGGGTATCACAATAT CGAGCAGGC
*CA-CaRop1*^a^	DQ257288	GTGGGTGATG**T**TGCTGTTGGC	CGCCAACAGCA**A**CATCACCCAC
*DN-CaRop1*^a^	DQ257288	GTTGGCAAGA**A**TTGTTT ATTG	CAATAAACAA**T**TCTTGCCAA C
*NtHSR201*	X95343	CAGCAGTCCTTTGGCGTTGT C	GCTCAGTTTAGCCGCAGTTG TG
*NtNPR1*	U76707	GGCGAGGAGTCCGTTCTTTAA	TCAACCAGGAATGCCACAGC
*NtPR2*	M60460	TGATGCCCTTTTGGATTCTATG	AGTTCCTGCCCCGCTTT
*NtACS1*	X65982	CATTAGCGAGGATTCGGAGTT	GTGGTGAATG AGGGATAGGA
*NtPR1b*	X66942	AACCCATCCATACTATTCCTTG	GAGCCGCTAACCTATTGTCCC
*NtNPK1*	D26601	ATGCAGGATTTCATCGGCTCCGTTC	CAAGGACGAG AAGGCAGA
*NtPI-I*	Z12619	GACTATGGTGAAGTTTGCTCAC	CCAAATATAAGTGGAATACATGG
*NtPI-II*	EF408803	ATGGCTGTTCACAAAGTTAGTTTCC	GTTCTTAGCG GATACCTC
*NtTPI*	AF542547	TTGGAATGTCTATGCTTGT	CAACCCTAGACTTCTGGAGATCA
*NtLOX1*	X84040	GTTGAAGGTTCTATCTGGCAGTTGG	TGTTGCGATCACGAATGGCTCTA
*CaActin*	AY572427	AGGGATGGGTCAAAAGGATGC	GAGACAACACCGCCTGAATAGC
*NtEF1*a	D63396	TGCTGCTGTAACAAGATGGATGC	GAGATGGGGACAAAGGGGATT

*^a^Primers for construction of constitutively active (CA) and dominant-negative (DN) *CaRop1* variants, the mutation site is underlined. *CaRop1* primers were used for full-length *CaRop1* cloning, other primers were used for measuring relative expression levels of the corresponding genes.*

### Construction of Vectors

For OE and transient expression, the full open reading frames (ORFs) of *CaRop1*, CA-*CaRop1*, or DN*-CaRop1* in the entry vector pDONR207 were transferred into various gateway-compatible destination vectors. For OE vector construction, the ORFs of *CaRop1*, CA-*CaRop1*, or DN*-CaRop1* were transferred into the pK7WG2 destination vector containing the *CaMV*35S promoter by LR reaction (Invitrogen, Carlsbad, CA, USA) to yield pK7WG2-*CaRop1*, pK7WG2-CA-*CaRop1*, and pK7WG2-DN*-CaRop1*. For construction of the *CaRop1–GFP* chimeric gene, a gateway compatible vector pMDC83 was employed. The ORF of *CaRop1* without the termination codon was cloned at the N-terminus of intact *GFP* and in-frame to the *GFP*-coding sequences (Invitrogen).

### Expression of CaRop1-GFP in *N. benthamiana* and Subcellular Localization

The recombinant plasmid p35S::CaRop1-GFP and the control plasmid p35S::GFP(pMDC83) were transformed into *Agrobacterium tumefaciens* GV3101. The subcellular localization of CaRop1 was determined as reported in our previous work with CaWRKY40 (Dang et al., 2013). *A. tumefaciens* cells carrying the different constructs were centrifuged and resuspended in an infiltration buffer (10 mM MgCl_2_, 10 mM MES, and 100 μM acetosyringone. pH 5.7) at a final OD_600_ of 0.4. Samples were infiltrated into 4–5-week-old *N. benthamiana* leaves. GFP was visualized using a laser scanning confocal microscope (Leica TCS SP8; Mannheim, Germany) with an excitation wavelength of 488 nm and a 505–530-nm band-pass emission filter.

### Construction of OE Transgenic *CaRop1*, CA-*CaRop1*, and DN*-CaRop1* Tobacco Lines

Because pepper plants are recalcitrant to genetic transformation, tobacco, which belongs to the same Solanaceae family as pepper, was used as a suitable transformation system for OE of *CaRop1*, CA-*CaRop1*, and DN*-CaRop1* to investigate the function of CaRop1. The OE vectors pK7WG2-*CaRop1*, pK7WG2-CA-*CaRop1*, or pK7WG2-DN-*CaRop1* was introduced into *A. tumefaciens* GV3101 via electroporation and then transformed into *N. tabacum* K326 using a conventional leaf-disk transformation method. The transformants were regenerated in MS medium containing 75 mg L^-1^ kanamycin, and the regenerated T_0_ plants, which were confirmed by PCR with kanamycin-specific primers, were cultivated in a soil mix of peat moss and Perlite (2:1, v/v) in plastic pots in the growth room. The T_0_ plants were self-pollinated to generate the seeds for the T1 lines. Similarly, the seedlings of the T_1_ lines were selected by kanamycin, and the seeds of the T_2_ lines were developed by self-pollination of the plants of the T_1_ lines. Expression levels of *CaRop1*, CA-*CaRop1*, and *DN-CaRop1* in the tobacco transformants were measured using semi-quantitative RT-PCR compared to the K326 control plants. Plants of the T_2_ lines that exhibited high expression of CaRop1 and their CA and DN mutants were used for further experiments.

### Pathogen Cultivation, Inoculation, and Plant Immunity Assay

Growth and inoculation of *R. solanacearum* FJC100301 were performed as described previously (Shang et al., 2012). Cells of FJC100301 were cultured in potato sucrose agar (PSA) medium (200 g potato, 20 g sucrose, 3 g beef extract, 5 g tryptone, 1 L water) and shaken at 200 rpm in an incubator at 28°C for 36 h. Then, samples were homogenized in sterile 10 mM MgCl_2_, and the cell density was diluted to 10^8^ colony-forming units (CFU)/mL (OD_600_ = 0.8). For the disease resistance assay, 2-month-old seedlings of the transgenic lines and the K326 line were inoculated by infiltration of 10 μL of *R. solanacearum* suspension (OD_600_ = 0.8) into the third leaves from the top using a syringe with a needle. To test the effect of OE of the CA and DN mutants on the growth of *R. solanacearum*, inoculated leaves were harvested 72 h post-inoculation (hpi), and leaf disks 3 cm from the inoculation sites were collected and homogenized in 10 mM MgCl_2_ (three disks per sample, 7-mm diameter). The homogenate was plated on PSA medium at appropriate dilutions. After incubation at 28°C for 2 days, the colonies were counted (Moeder et al., 2005). For analysis of the hypersensitive response (HR), the inoculated leaves were harvested at 72 hpi, whole leaves were stained with trypan blue (Shang et al., 2012), and the typical phenotypes were photographed. The phenotypic effects of the OE of CaRop1 in the CA and DN mutants on the resistance of plants to pathogen inoculation were detected and photographed at 14 days post-inoculation (dpi). To analyze the transcriptional expression of defense-related genes in response to pathogen infection, the *R. solanacearum* inoculated third leaves from the top were harvested at 36 hpi for preparation of total RNA.

### Aphid Culture and Bioassays

Aphids used in this study were harvested in a tobacco field at an experiment station at FAFU. An aphid population (*Myzus persicae*) was maintained on K326 plants in a pesticide-free greenhouse. The 5-month-old plants from the T_2_ tobacco lines and the control line were artificially inoculated with 10 aphid larvae on the abaxial surface of the third leaf from the top and were placed in a random arrangement in the pesticide-free greenhouse. The aphid population on each plant was photographed and counted 15 dpi. At least three plants of each line were examined.

### Total RNA Isolation and Quantitative Real-Time RT-PCR

Total RNA was isolated with the TRIzol reagent (Invitrogen) following the manufacturer’s instructions. The RNA sample was then reverse transcribed using the PrimeScript^TM^ RT-PCR kit in a 10-μl volume. The resulting cDNA was diluted 10-fold and then amplified using SYBR^®^ Premix Ex Taq^TM^ II (TaKaRa Perfect Real Time; Dalian, China) using an Applied Biosystems 7500 Real-Time PCR system. Reactions were conducted in 10-μL volumes under the following conditions: 95°C for 30 s; 40 cycles of 95°C for 5 s followed by 60°C for 34 s; 95°C for 15 s; 60°C for 1 min; 95°C for 15 s; and 60°C for 15 s. Amplification of the target genes was monitored each cycle with SYBR-green fluorescence. The *C*t (threshold cycle), which is defined as the real-time PCR cycle at which a statistically significant increase of reporter fluorescence is first detected, was used as a measure of the starting copy number of the target gene. Five replicates of each experiment were performed, and normalized transcript level data for the target genes were analyzed by qPCR and the Livak method (Livak and Schmittgen, 2001). Relative expression levels of pepper target genes were normalized to the expression of *CaActin*, and the relative expression levels of target genes in transgenic tobacco were normalized to the expression of *NtEF1α.*

### Histochemical Staining

Staining with trypan blue was performed according to the previously published method of Cai et al. (2015).

## Results

### Cloning and Sequence Analysis of *CaRop1* cDNA

**FIGURE 2:**
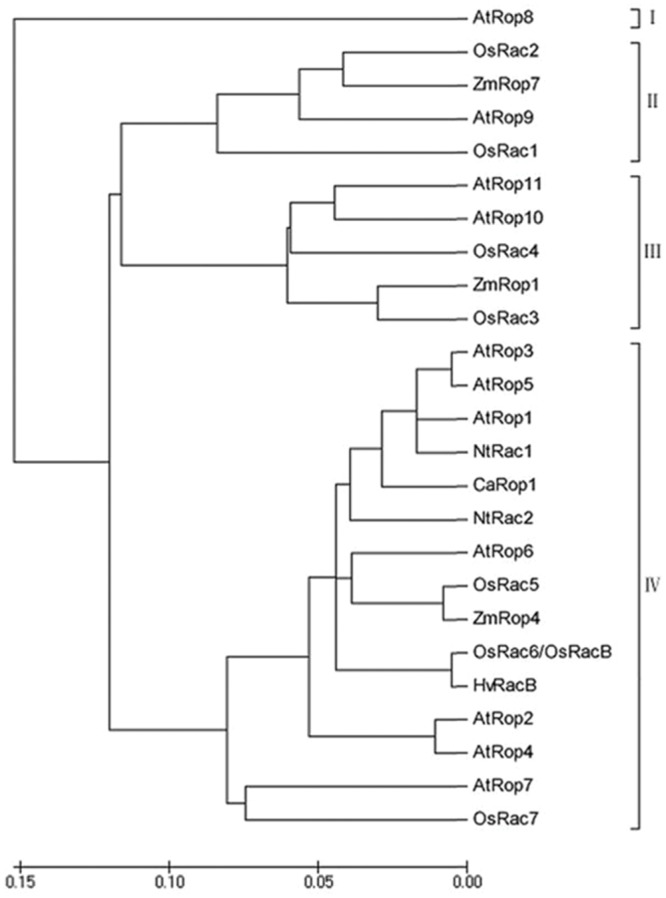
**Phylogenetic Analysis of Rop/Rac GTPases.** Phylogenetic tree for the deduced amino acid sequences of Rop GTPases from *Arabidopsis*, rice, and pepper was constructed using NJplot. Abbreviations for species: At, *Arabidopsis thaliana*; Os, *Oryza sativa*; Ca, *Capsicum annuum*; Zm, *Zea mays*; Hv, *Hordeum vulgare*; Nt, *Nicotiana tabacum*. Accession numbers: AtRop1-AtRop11 are At3g51300, At1g20090, At2g17800, At1g75840, At4g35950, At4g35020, At5g45970, At2g44690, At4g28950, At3g48040; and At5g62880. OsRac1-OsRac7 are Q9SSX0, Q68Y52, Q6Z808, Q67VP4, Q6EP31, Q6ZHA3, and Q6Z7L8; *ZmRop1*, *ZmRop4*, and *ZmRop7* are NP_001104929, NP_001105719, and NP_001105523; *HvRacB* is AJ344223; *NtRac1*and *NtRac2* are AAK31299 and AAD00118; and *CaRop1* is DQ257288. Roman numerals I-IV refer to the four subgroups of Rops.

To acquire full-length cDNAs of Rop family members in *C. annuum*, ESTs of *C. annuum* that are homologous to *AtRac1* were searched using BLASTN with AtRac1 as a query sequence, and the acquired ESTs were assembled into contigs. A contig with high sequence similarity to *AtRac1* was selected, and a specific pair of primers was designed for use in a PCR-based 96-well screen of a cDNA library of pepper leaves. A positive clone was acquired after three rounds of screening, and sequencing revealed that this clone was a full-length cDNA, which was designated as *CaRop1* (GenBank Accession Number ABB71820). *CaRop1* is 1,149 bp in length and harbors an ORF that encodes a 197-amino acid polypeptide with a conserved Rho domain with a predicted molecular mass of 21,443.7 kD and a pI of 9.3. Multiple alignment analysis showed that CaRop1 has a high sequence similarity to Rops of other plant species and contains all the structural features of a type-I plant Rop. This conclusion was based on the presence of the C-terminal canonical CaaL motif, which is required for prenylation and membrane attachment (Agrawal et al., 2003; Wu et al., 2011). A phylogenetic tree constructed from CaRop1 and Rops from other plant species revealed that CaRop1 belongs to a subgroup that also contains HvRacB, OsRacB, AtRop1, and AtRop6. All of these Rop proteins have been implicated in plant responses to attack by pathogens (**Figure 2**) (Schultheiss et al., 2002, 2003; Poraty-Gavra et al., 2013). Based on the tenet of phylogeny of function, that is, that genes of similar function are likely to be grouped in a phylogenetic tree (Pereira-Leal and Seabra, 2001), we speculate that CaRop1 participates in pathogen-response signaling.

### Subcellular Localization of CaRop1

**FIGURE 3:**
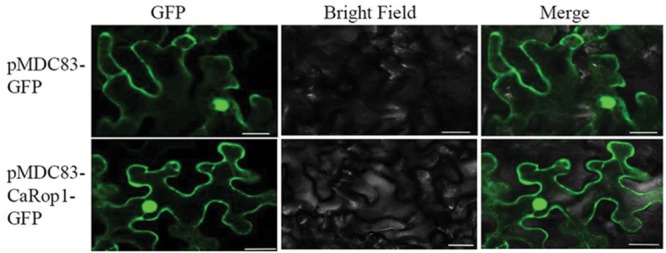
**Subcellular localization of *Ca*Rop1 in *Nicotiana benthamiana* leaf epidermal cells.** The CaRop1-GFP fusion protein localized at the plasma membrane, cytoplasm, and nucleus of *N. benthamiana* leaf epidermal cells. GFP alone was found throughout the cells. Images of GFP were obtained using a laser scanning confocal microscope with an excitation wavelength of 488 nm and a 505–530-nm band-pass emission filter.

To determine the subcellular localization of CaRop1 *in vivo*, the *CaRop1* ORF without its termination codon was fused to intact GFP-coding sequences at the N-terminus. The localization pattern of CaRop1-GFP was analyzed in transient expression experiments using *N. benthamiana* leaf epidermis. Generally, GFP signals were observed throughout the *N. benthamiana* leaf epidermal cells in the plasma membrane, cytoplasm, and nucleus (**Figure 3**) in agreement with a previous observation that type I Rops in rice were present throughout the cell (Chen et al., 2010b).

### Susceptibility of Tobacco to *R. solanacearum* Infection Was Reduced by OE of *CaRop1* and DN-*CaRop1* but Enhanced by OE of CA-*CaRop1*

**FIGURE 4:**
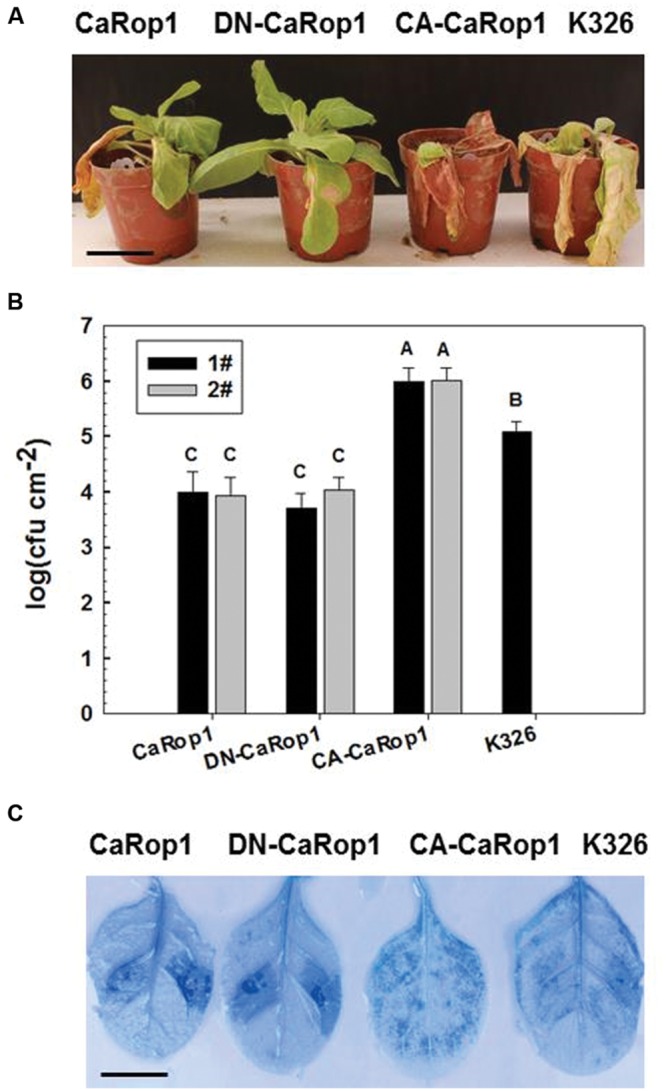
**HR-associated cell death was triggered by overexpression of CA-CaRop1, DN-CaRop1, and CaRop1 in plants. (A)** The phenotype of the CA-CaRop1, DN-CaRop1, and CaRop1 OE in tobacco plants after inoculation of *R. solanacearum* at 14 dpi is shown. Tobacco plants were equivalently inoculated with a concentration of 1 × 10^8^ CFU/mL of bacteria in the lateral veins of the third leaves from the top. Three plants for each line were tested, and the phenotypes of the plants were photographed at 14 dpi. **(B)** The effect of overexpression of CA-CaRop1, DN-CaRop1, and CaRop1 on the growth of *R. solanacearum*. Leaf disks 3 cm away from the inoculated site were cut at 72 hpi, and the CFU/mL of *R. solanacearum* per sample (each from three leaf disks) were measured. The values are shown as mean ± SD of samples from three independent plants. The experiments were repeated three times with two lines for each construct. Different letters indicate statistically significant differences (Student–Newman–Kuels test; *P* < 0.01). **(C)** Trypan blue staining of the leaves of tobacco plants was performed 3 dpi of the third leaves from the top with 10 μL of *R. solanacearum* suspension (OD_600_ = 0.8) using a syringe with a needle. Scale bars = 1 cm.

To assay the possible function of CaRop1 in plant immunity, we generated transgenic tobacco lines that overexpressed CaRop1 or its CA or DN mutant. At least ten independent transgenic T_2_ tobacco lines were obtained for each genotype, and none of these lines exhibited any significant phenotypic difference or growth retardation compared to the wild-type K326 plants. Two T_2_ lines of each genotype were randomly chosen for further assay. These lines were challenged by *R. solanacearum*, a soil-borne bacterium that causes lethal disease by inducing wilting symptoms in more than 200 plant species, including economically important crops, such as pepper and tobacco. A highly virulent strain of *R. solanacearum* FJC100301 (Dang et al., 2013) was used to inoculate the plants of transgenic tobacco and the control. At 7 dpi, obvious wilting of leaves of K326 and CA-*CaRop1*-OE plants was observed, whereas only faint wilting was found in leaves of *CaRop1-* and DN-*CaRop1*-OE lines. At 14 dpi, extremely severe decaying symptoms were found in CA-*CaRop1* and K326 plants, but the *CaRop1*- and DN-*CaRop1*-OE lines exhibited only limited lesions (**Figure 4A**). Consistently, higher CFU were detected in *R. solanacearum*-inoculated CA-*CaRop1* lines and K326 plants compared to those in *R. solanacearum*-inoculated *CaRop1* and DN-*CaRop1* tobacco lines (**Figure 4B**). As HR cell death has been previously found to be a hallmark in plant immunity, especially in effector-triggered immunity (Jones and Dangl, 2006), HR cells in different tobacco transgenic plant lines were investigated. As evidenced by trypan blue staining, consistent HR cell death was observed in inoculated leaves of *CaRop1*- and DN-*CaRop1*-OE lines at 3 dpi, while little or no HR cell death was noted in the inoculated leaves of the control or CA-lines, respectively (**Figure 4C**).

**FIGURE 5:**
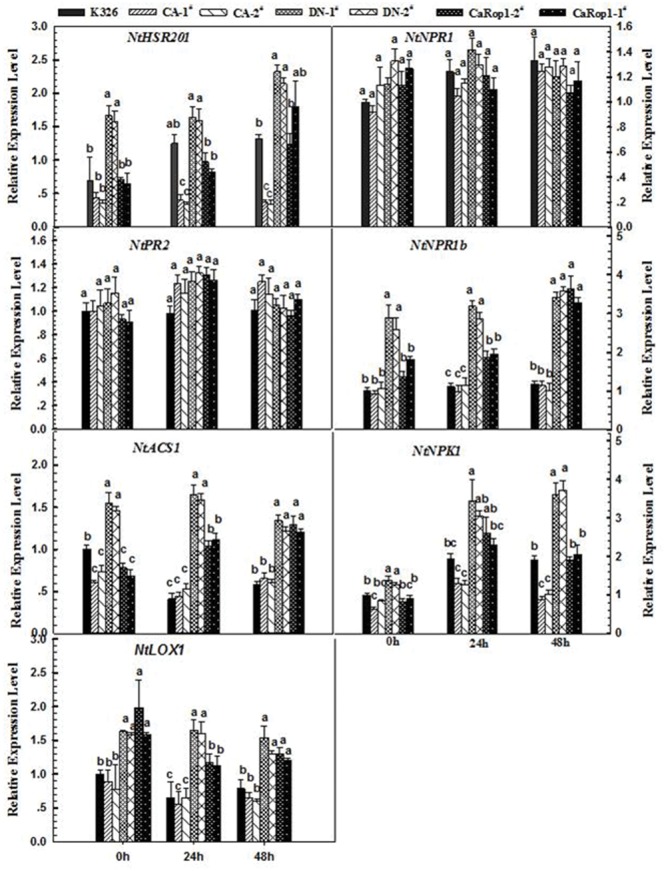
**Immunity-associated marker genes were upregulated by OE of CaRop1 and DN-CaRop1 but were downregulated by overexpression of CA-CaRop1.** Tobacco plants were equivalently inoculated with a concentration of 1 × 10^8^ CFU/mL of bacteria in the lateral vein of the third leaves from the top, which were harvested at 36 hpi for preparation of RNA to be used as a template for real-time RT-PCR with specific primer pairs of immunity-associated marker genes, including *NtHSR201*, *NtNPR1*, *NtPR2*, *NtPR1b*, *NtLOX1*, *NtACS1*, and *NtNPK1*. Defense-related gene transcript levels of wild-type tobacco K326 were used as a reference, which was set as “1.” Each value is given as the average of three replicate experiments ±SD. Different letters indicate statistically significant differences (Student–Newman–Kuels test; *P* < 0.05).

To further elucidate the possible mode of action of *CaRop1*-mediated defense, transcriptional responses of known defense-associated marker genes were investigated in two independent CA-, DN- CaRop1-, and CaRop1-OE transgenic lines using qPCR. Defense-associated genes examined in this study include the HR-associated gene *NtHSR201* (Takahashi et al., 2004), the SA-responsive genes *NtPR2* and *NtNPR1* (Spoel et al., 2003; Sohn et al., 2007), the ET production-associated gene *NtACS1* (Sohn et al., 2007), the JA-responsive gene *NtPR1b* (Sohn et al., 2007), the JA biosynthesis-associated gene NtLOX (Halitschke and Baldwin, 2003*)*, and *NPK1*, a MAPKKK gene involved in plant immunity (Jin et al., 2002). All of these genes were previously identified to be upregulated in response to *R. solanacearum* infection in tobacco (Dang et al., 2013). The transcript abundances of *NtHSR201*, *NtACS1*, *NtPR1b, NtLOX*, and *NtNPK1* were higher at 36 hpi n *R. solanacearum*-inoculated plants of *CaRop1* and DN-*CaRop1* lines than in CA-*CaRop1* lines and K326 plants, while transcriptional levels of *NtNPR1* and *NtPR2* did not differ notably among the tested genotypes (**Figure 5**).

### Susceptibility of Tobacco to Aphid Attack Was Reduced by OE of DN-*CaRop1* or *CaRop1* but Was Enhanced by OE of CA-*CaRop1*

**FIGURE 6:**
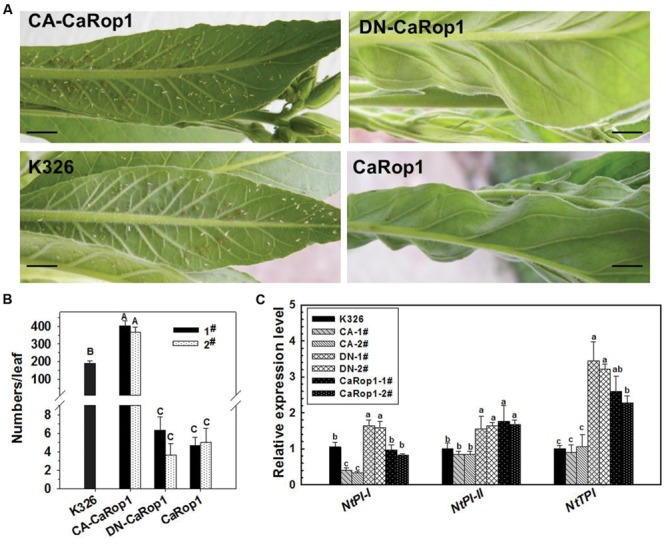
**The effect of overexpression of DN-CaRop1, CA-CaRop1, and CaRop1 on the resistance of tobacco plants to aphids and on the transcriptional expression of *NtPI-I, NtPI-II* and *NtTPI*. (A)** The aphid population on the artificially inoculated leaves of 5-month-old seedlings of CaRop1, DN-CaRop1, CA-CaRop1 overexpressing transgenic lines or the wild type K326 at 15 dpi. **(B)** The number of aphids on each artificially inoculated leaf of 5-month-old tobacco seedlings of different genotypes at 15 dpi. **(C)** Transcriptional expression of *NtPI-I*, *NtPI-II*, and *NtTPI* in 5-month-old DN-*CaRop1*, CA-*CaRop1* and *CaRop1* OE tobacco seedlings was determined by real-time RT-PCR. Transcript levels of the marker genes in K326 were used as a reference and were set as “1.” Each value is the average of three replicate experiments ±SD, and different letters indicate statistically significant differences (Student–Newman–Kuels test; *P* < 0.05). Scale bars = 1 cm.

Plants of T_2_ tobacco lines and K326 tobacco were exposed to aphids in a greenhouse. The plants were attacked by the aphids, and large numbers colonized the abaxial surface of the leaves. Interestingly, the aphid population success varied considerably in different plant genotypes. CA-*CaRop1* obviously facilitated the colonization and reproduction of aphid larva on the leaves, while DN-*CaRop1* and *CaRop1* strongly lowered aphid colonization. To confirm the defensive role of *CaRop1* in aphid response, we repeated the aphid infestation experiment by artificial inoculation. Briefly, aphid adults (10 days) were transferred from leaves of nursery K326 seedlings to experimental plants with a fine toothpick. Fifteen days later, the number of aphids on each inoculated plant was assessed, and the typical phenotypes were photographed. Consistently, large numbers of aphids were found in the control plants and CA-CaRop1-OE plants, whereas, no aphids or only small numbers of aphids were found in the DN-CaRop1- and CaRop1-OE plants (**Figures 6A,B**).

To further investigate the role of CaRop1 in the response of tobacco plants to aphid attack and to elucidate the possible mechanism, we examined the effect of OE of *CaRop1* on the transcript levels of *NtPI-I, NtPI-II*, and *NtTPI* in two independent transgenic lines (**Figure 6C**). Results of qPCR showed that the transcriptional expression of *NtPI-I* was significantly triggered by OE of *CaRop1* or DN-*CaRop1* but that this expression was downregulated or remained unchanged by OE of CA-*CaRop1* in tobacco plants compared to that in the wild-type plants. On the other hand, the transcriptional expression of *NtPI-II* and *NtTPI* were significantly upregulated in *CaRop1-* or DN-*CaRop1-OE* tobacco plants but remained unchanged in CA-*CaRop1-OE* tobacco plants compared to that in the wild-type plants.

## Discussion

The Rop/Rac family is highly conserved in the plant kingdom. To date, seven Rop/Rac family members have been identified in rice (Miki et al., 2005), and 11 have been identified in *Arabidopsis* (Zheng and Yang, 2000). The induced amino acid sequence of our newly identified *CaRop1* contains a conserved Rho domain and has high sequence similarity to type I Rops, such as *AtRop1* and *AtRop6* in *Arabidopsis* and *OsRacB* in rice. Some type I Rops have been reported to localize to the plasma membrane, the cytoplasm, and the nucleus, due to their C-terminal regions (Bischoff et al., 2000; Schultheiss et al., 2003; Xiao et al., 2009; Chen et al., 2010b). This localization has been reported for OsRac5, OsRacB/OsRac6, and OsRac7 in rice; AtRop4 and AtRop6 in *Arabidopsis*; and HvRacB in barley (Bischoff et al., 2000; Schultheiss et al., 2003; Xiao et al., 2009; Chen et al., 2010b). Not surprisingly then, CaRop1, which also contains the conserved C-terminal canonical CaaL motif found in other type I Rops, was also localized to the nucleus, the cytoplasm, and plasma membrane. This similarity with other type 1 Rops suggests that CaRop1 is a type I Rop of *C. annuum.* Among the Rop/Rac family of proteins in plant species, differences with respect to structure, function, and expression have been described. The 11 *Arabidopsis* Rops are divided into four phylogenetic groups with distinct functions (Gu et al., 2004; Hou et al., 2011), and the seven members of the Rop family in rice differ in their expression patterns (Xiao et al., 2009).

In our study, OE of *CaRop1* and DN-*CaRop1* enhanced resistance of the transgenic tobacco plants to *R. solanacearum* inoculation, while OE of CA-*CaRop1* actually enhanced susceptibility to this pathogen. The altered resistance of these transgenic plants was accompanied by transcriptional modification of *NtHSR201*, *NtACS1*, *NtPR1b*, and *NtLOX1*, which are genes that have been implicated previously in plant immunity (Czernic et al., 1996; Jin et al., 2002; Sohn et al., 2007; Gao et al., 2008; Borges et al., 2012; Dang et al., 2013; Nalam et al., 2015). These changes are consistent with our finding that immunity against *R. solanacearum* was altered by the ectopic expression of CaRop1 as well as by expression of DN and CA mutant CaRop1. Similarly, plants expressing a DN form of *AtRop6* exhibit a gene expression profile associated with constitutive SA-mediated defense responses and enhanced pre-invasive defense responses to a host-adapted virulent powdery mildew fungus (Poraty-Gavra et al., 2013). In addition, ectopic expression of CA forms of *HvRACB*, *HvRAC3*, and *HvRop6* in barley enhance susceptibility of transgenic plants to powdery mildew (Schultheiss et al., 2003, 2005). Since these Rops are grouped together in a phylogenetic tree (**Figure 2**), conservation of their function may also correlate with their structurally similarity. Furthermore, our results are also consistent with findings that RanGAP2, a GAP that mediates the transformation of GTP-bound Rop to GDP-bound Rop, acts as positive regulator of resistance of potato to virus X via interaction with the resistance protein Rx (Sacco et al., 2007; Tameling and Baulcombe, 2007; Rairdan et al., 2008; Tameling et al., 2010; Sturbois et al., 2012). In contrast, OE of a CA-*OsRac1* induces cell death, and OE of a DN-*OsRac1* blocks hydrogen peroxide production and cell death in transgenic lesion-mimic mutants (Fujiwara et al., 2006). Together, these results suggest that members of the Rop family in different plant species play important roles in plant immunity via different modes of action.

We also found that OE of CA-*CaRop1* enhances the population size of aphid nymphs on leaves of inoculated transgenic tobacco, whereas OE of DN-*CaRop1* or *CaRop1* decreases the population size. Transcriptional expression of *NtPI-I, NtPI-II*, and *NtTPI*, which encode tobacco protease inhibitors, were previously found to be upregulated by methyl jasmonate (MeJA) vapors, wounding, and attack by *Manduca sexta* larvae in tobacco plants and are probably involved in insect resistance (Anderson et al., 1997; Rocha-Granados Cdel et al., 2005; Maheswaran et al., 2007; Srinivasan et al., 2009). Expression of these genes was triggered in transgenic tobacco by OE of DN-*CaROP1*; although, these levels were decreased or remained unchanged by OE of CA-*CaRop1*, suggesting that *CaRop1* acts as a regulator in the response of plants to aphid attack. Partial overlap between defense signaling pathways against herbivores and microbial pathogens has been reported previously (De Vos et al., 2005, 2007). For example, responses to aphid attack at the proteome level are broadly similar to basal non-specific defense and stress responses in wheat (Ferry et al., 2011). Defense-associated pathogenesis-related responses and calcium-dependent signaling in wheat are also induced by the Russian wheat aphid 2 (Botha et al., 2010). Treating seeds with activators of plant defense, such as JA or β-aminobutyric acid, generate long-lasting priming of resistance to aphids as well as to the necrotrophic fungal pathogen *Botrytis cinerea* (Worrall et al., 2012). Additionally, signaling cascades, such as those related to alpha-DIOXYGENASE1, Hsp90, Sgt1, PAD4, SlSERK1, and MAPK, and to phytohormones, such as SA, JA, and ET, have also been implicated in plant responses to both pathogen and aphid attack (Zhou et al., 1998; Hamberg et al., 2003; Mantelin et al., 2011).

Together, these results suggest extensive crosstalk between the plant response to a pathogen and herbivore attack, possibly implicating the JA and ET pathways, which are shared in plant responses to pathogens and herbivores (Glazebrook, 2005; Zavala and Baldwin, 2006; Chehab et al., 2008; Howe and Jander, 2008; Simons et al., 2008; Demkura et al., 2010). In the present study of the functional characterization of CaRop1 using transgenic plants, CA-CaRop1, DN-CaRop1, and CaRop1 all significantly modulated the response of pepper to *R. solanacearum* and aphid attack, accompanied with transcriptional modulation of JA-dependent *PR1b* and ET-dependent *ACS1.* On the other hand, however, transcription of SA-dependent *PR2* and *NPR1* were not altered by *CaRop1*and its CA or DN mutant. These findings strongly suggest that the modulation of defense against *R. solanacearum* attack by *CaRop1* is at least partially associated with JA and ET-dependent signaling machinery.

Overexpression of *CaRop1* also decreased susceptibility of transgenic tobacco plants to *R. solanacearum* and aphid attack in a manner similar to that of DN-CaRop1. Upon inoculation with *R. solanacearum*, the mRNA levels of *NtHSR201*, *NtPR1b*, and *NtACS1* in plants of the DN-CaRop1 lines were significantly higher than those in the K326 plants at 36 hpi. Similarly, upon aphid attack, *NtPI-I*, *NtPI-II*, and *NtTPI* were significantly enhanced by OE of CaRop1. We suggest that some components, perhaps GAPs, are induced by pathogen infection, and this induction, in turn, regulates the conversion of GTP-bound CaRop1 to GDP-bound CaRop1. Another important component that may be involved in *CaRop1*-mediated crosstalk between responses in pepper to *R. solanacearum* and to aphid attack is NPK1, a MAPKKK that was previously found to interfere with the function of the disease-resistance genes *N*, *Bs2*, and *Rx* (Jin et al., 2002). Our data show that *NPK1* is transcriptionally induced by OE of DN-*CaRop1* and by *CaRop1* but that this transcription is decreased by OE of CA-*CaRop1*, suggesting that NPK1 acts as a downstream component in the CaRop1-mediated defense signaling pathway.

Collectively, our data demonstrate that the ectopic expression of CaRop1 modulates the response of tobacco plants to *R. solanacearum* and to aphid attack, possibly via the JA and ET signaling pathways. Furthermore, we speculate that CaRop1 may play a role in the crosstalk between the Solanaceae response to pathogen and insect attack. Further confirmation of the role of CaRop1 in pepper plants and identification of the effectors of CaRop1 or its direct downstream signaling components will likely provide new insight into the molecular mechanisms underlying CaRop1-mediated synergistic resistance to *R. solanacearum* and aphid attack.

## Author Contributions

AQ and SH conceived and designed research. AQ and ZL conducted primary experiments. JL, YC, and DG performed replication genotyping with the transgenic plants. AQ and ZL contributed comments during manuscript preparation. DG contributed new reagents or analytical tools. SH and AQ performed expression analysis and editing of the manuscript. All authors read and approved the manuscript.

## Conflict of Interest Statement

The authors declare that the research was conducted in the absence of any commercial or financial relationships that could be construed as a potential conflict of interest.

## Footnotes

1 http://blast.ncbi.nlm.nih.gov/Blast.cgi
